# Prenatal detection of a 3q29 microdeletion in a fetus with ventricular septum defect: A case report and literature review

**DOI:** 10.1097/MD.0000000000024224

**Published:** 2021-01-08

**Authors:** Fagui Yue, Shu Deng, Qi Xi, Yuting Jiang, Jing He, Hongguo Zhang, Ruizhi Liu

**Affiliations:** a Center for Reproductive Medicine, Center for Prenatal Diagnosis, First Hospital; b Jilin Engineering Research Center for Reproductive Medicine and Genetics, Jilin University, Changchun, China.

**Keywords:** 3q29 microdeletion, genetic counseling, single nucleotide polymorphism array, ventricular septum defect

## Abstract

**Rationale:**

Chromosomal 3q deletion is a recurrent genomic alternation, which is rarely reported in clinic.

**Patient concerns:**

A 27-year-old woman underwent amniocentesis for cytogenetic analysis and single nucleotide polymorphism (SNP) array analysis at 27 weeks of gestation, due to ventricular septum defect in prenatal ultrasound findings.

**Diagnoses:**

G-banding analysis showed the karyotype of the fetus was normal and the couple also had normal karyotypes. However, SNP array detected a 1.71 Mb microdelection in 3q29, which was described as arr[hg19]3q29(194184392–195887205) × 1. There are 12 genes located in this locus.

**Interventions:**

The couple refused SNP array to testify the 3q29 microdeletion was inherited or *de novo* and they chose termination of pregnancy.

**Outcomes:**

The deleted region in the fetus overlapped with part 3q29 microdeletion syndrome, which was characterized by learning disability, speech delay, mental deficiency, ocular abnormalities and craniofacial features. In addition, no similar/overlapping 3q29 microdeletion cases were reported according to the published literature and database.

**Lessons:**

For the chromosomal microscopic imbalances partially overlapping with the defined pathogenic syndrome, deleted/duplicated size, genetic materials and phenotypic diversity should be taken into consideration when genetic counseling is offered by the clinicians.

## 1 Introduction

Chromosomal microscopic imbalances, microdeletions/microduplications, are associated with multiple genetic disorders, including intellectual disability (ID), developmental delay, autistic spectrum disorders (ASD) and congenital abnormalities.^[1]^ For submicroscopic copy number variations (CNVs) which are too small to be detected by banding technique, chromosomal microarray analysis (CMA) allows the detection of these chromosomal aberrations associated with well-described phenotypes.^[2]^ With the extensive application of high-resolution microarrays, a large number of microdeletions and microduplications syndromes have been described in recent years, with 22q11.2, 7q11.23, 17p11.2 and 16p11.2 most common in clinic.^[1,3]^ Meanwhile, pathogenic CNVs, due to deletions or duplications, may lead to variable expressivity among individuals.^[4]^

Terminal deletion of 3q, mostly *de novo*, was a recurrent genomic alternation which was first reported in 2001.^[5]^ The incidence rate of 3q29 deletion was approximately 1/30,000-40,000.^[6]^ Till now, more than 40 cases involving submicroscopic 3q29 deletion have been reported in published literature.^[7]^ The clinical manifestations were variable and characterized by learning disability, mental deficiency, speech delay, ocular abnormalities and craniofacial features, such as high nasal bridge and microcephaly. In addition, autism, heart defects, hypospadias and gastrointestinal abnormalities were also observed in some cases.^[7,8]^ Generally speaking, there might exist phenotypic diversity in patients with 3q29 microdeletion.

In prenatal cases with normal karyotypes, the detection rate of chromosomal microdeletion/microduplication was approximately 1% of pregnancies without structural abnormalities and 6% with structural abnormalities.^[2]^ Herein, we report a fetus with a 1.7 Mb microdeletion in chromosome 3q29, accompanied with abnormal prenatal ultrasound findings. We have also provided a literature review on cases with similar 3q29 microdeleted locus involving cardiac defect phenotypes.

## 2 Methods

The study protocol was approved by the Ethics Committee of the First Hospital of Jilin University (No.2017–441), and written informed consent was obtained from the couple for publication of this case report and accompanying images.

### 2.1 Cytogenetic analysis

Amniocentesis was performed for karyotyping analysis with informed consent. The sample was collected from amniotic fluid cells, and was cultured by standard operating procedure. Cultured peripheral blood cells of the couple were also obtained for karyotyping. Cytogenetic analysis was performed by G-banding technique in 20 metaphases for all samples, with a resolution of 300–400 bands. Chromosomal karyotypes were described based upon the International System for Human Cytogenetic Nomenclature.^[9]^

### 2.2 Single nucleotide polymorphism (SNP) array

SNP array analysis was carried out using the Human CytoSNP-12 DNA Analysis BeadChip (Illumina, San Diego, CA). Genomic DNA was extracted from 10 mL uncultured amniotic fluid cells using QIAamp DNA Mini kit (Qiagen, Hilden, Germany). Data was analyzed by Illumina's Genome Studio software. The final results were analyzed using Database of Chromosomal Imbalance and Phenotype in Humans using Ensemble Resources, Database of Genomic Variants, Online Mendelian Inheritance in Man (OMIM) and the National Center for Biotechnology Information.^[10]^

## 3 Case presentation

A 27-year-old, gravida 2, para 0, woman accepted amniocentesis for cytogenetic and SNP array analysis at 27-week gestation due to 27 weeks’ sonography findings inferring ventricular septum defect (VSD) at the Center for Reproductive Medicine and Center for Prenatal Diagnosis. The wife and her husband were nonconsanguineous and healthy. There was no family history of diabetes mellitus or congenital malformations. The mother denied any exposure to alcohol, teratogenic agents, irradiation, or infectious diseases during this pregnancy.

**Figure 1:**
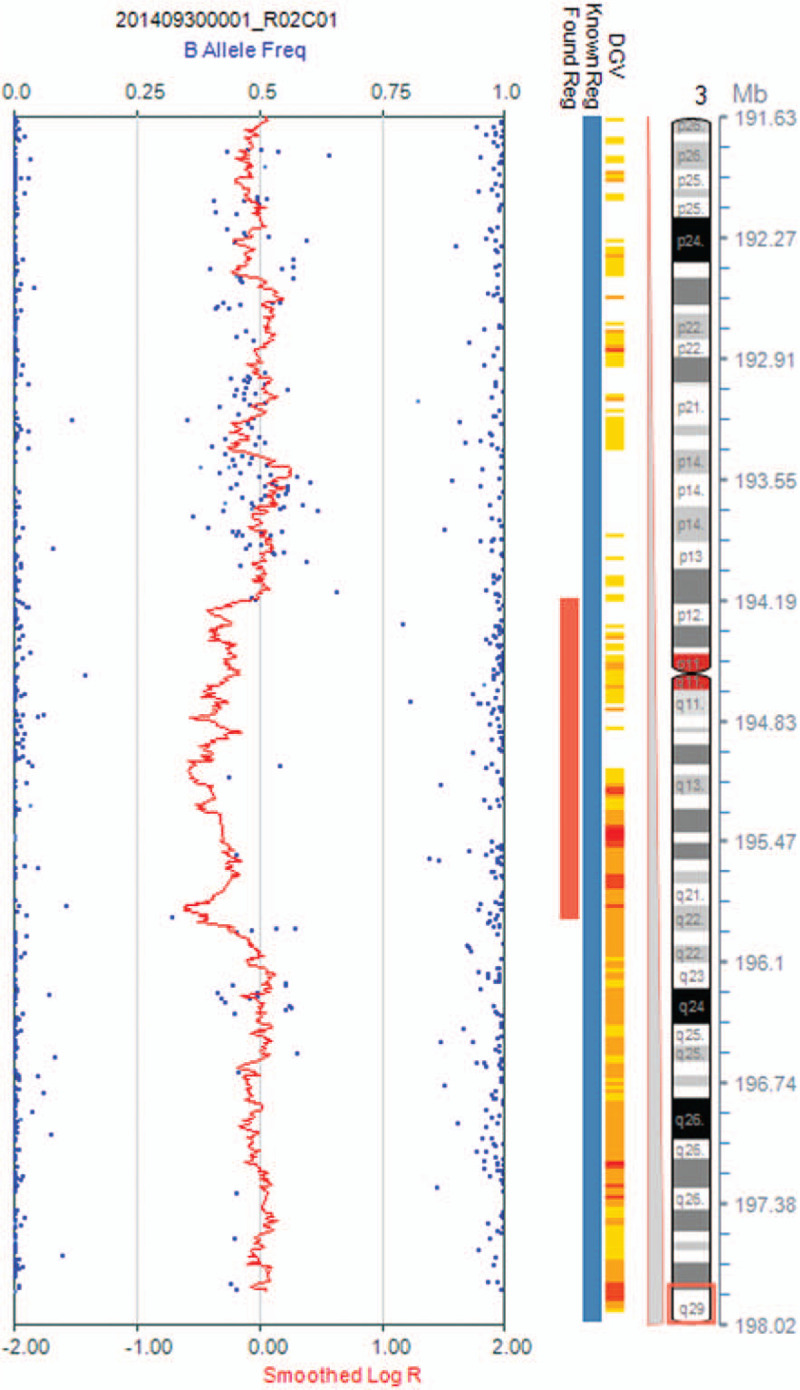
SNP array on uncultured amniocytes depicted 3q29 deletion.

**Figure 2:**
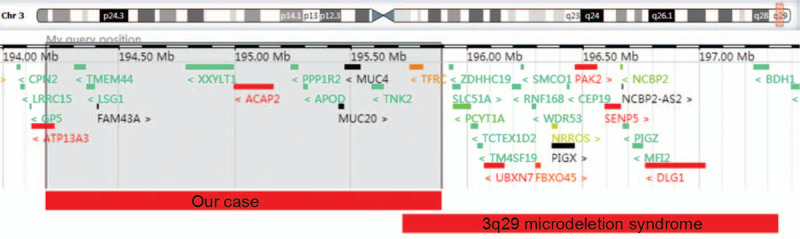
The involving genes contained in our case with 3q29 microdeletion (chr3:194184392-195887205) and typical region of 3q29 microdeletion syndrome. This figure is modified from DECIPHER genome browser. DECIPHER = Database of Chromosomal Imbalance and Phenotype in Humans using Ensemble Resources.

The fetus presented ventricular septum defect in ultrasound examination. G-banding analysis described the chromosomal karyotype of fetus as normal. However, SNP analysis identified a 1.71 Mb deletion in 3q29: arr[hg19]3q29 (194184392–195887205) × 1 (Fig. 1), overlapping with part 3q29 microdeletion syndrome. In our case, 12 genes are located in this deleted region, including *ATP13A3, TMEM44, LSG1, FAM43A, XXYLT1, ACAP2, PPP1R2, APOD, MUC20, MUC4, TNK2* and *TFRC* (Fig. 2). Chromosomal karyotypic analysis showed that the couple presented normal karyotypes. The couple refused to accept SNP analysis to identify the 3q29 microdeletion in the fetus was *de novo* or parentally inherited. According to genetic counseling, the couple finally chose to terminate the pregnancy based upon abnormal SNP results and ultrasound abnormalities.

## 4 Discussion

In our study, we delineated a rare prenatal case presenting VSD in ultrasound findings, who carried a 1.71 Mb deletion in the region of 3q29 (chr3: 194184392–195887205, hg19) using cytogenetic and molecular cytogenetic techniques. To the best of our knowledge, no similar 3q29 microdeletion cases in published literature and databases were reported before.

The 3q29 microdeletions are rare recurrent CNVs, most of which are *de novo*.^[8]^ This syndrome is associated with a wide range of clinical features among the individuals, mainly characterized by intellectual disability, development delay, language retardation, craniofacial abnormalities, musculoskeletal abnormalities, autism, and congenital heart defect. According to the clinic observation, patients with this subchromosomal deletion in the terminal of 3q are usually discovered based upon clinical findings in childhood, with typically heterozygous 1.6 Mb deletion.^[11]^

Till now, cases with 3q29 microdeletion are rarely reported in clinic.^[6–8,12]^ Considering the unusual cardiac defect discovered in such cases, we reviewed the literature and made a comparison on 3q29 microdeletion cases presenting cardiac anomalies to delineate the phenotype-karyotype correlations clearly (Table 1).^[7,13–18]^ All 3q29 microdeletions varied in size, from 0.96 Mb to 1.6 Mb. The age of the cases ranged from 10 months to 13 years: 3/10 cases were *de novo*, 3/10 cases were parentally inherited, and this detail was not available in 4/10 cases. The clinical characteristics were as follows: congenital heart defect (9/10), development delay (6/10), mental retardation (6/10), craniofacial dysmorphic features (6/10), which were consistent with previous reports of 3q29 microdeletions. Diverse facial dysmorphisms could also be discovered in these cases: Ear anomalies (4/10), microcephaly (3/10), short philtrum (3/10), abnormal nasal bridge (2/10). In addition, gastroesophageal signs was observed in 2/10 cases. Based upon the observations above, a complex of other clinical abnormalities existed in these 3q29 microdeletion cases presenting with cardiac disease. The deleted locus in our case overlapped with part region of typical 3q29 duplication syndrome, which might be responsible for the abnormal ultrasonagraphy to some extent. In addition, some rare anomalies involving neural and skeletal development have been reported in some cases. Guo et al^[12]^ described a 7-year-old girl with Chiari malformation type II and Sprengel's deformity, accompanied by a novel 666 kb microdeletion in 3q29 (chr3:194,532,035–195,198,585; hg19). Since the couple in our study did not accept SNP array, we fail to confirm the deletion was *de novo* or inherited. They made the decision to terminate the pregnancy, so we could not predict the postnatal growth conditions of the fetus in future. Hence irrespective of whether the 3q29 microdeletion is typical or atypical, more clinic data is required to establish a clear phenotype-karyotype correlation.

**Table 1:** Literature review of 3q29 microdeletion with congenital heart defect.

	Chirita Emandi et al^[7]^	Citta et al^[13]^	Dasouki et al^[14]^	Digilio et al^[15]^	Li et al^[16]^			Monfort et al^[18]^		
References	Patient 1	Patient 3	Patient 2	Patient 3	Patient 1	Patient 1 father	Ballif et al^[17]^	Patient 1	Patient 1 mother	Our case
Gender	M	M	F	F	M	M	NA	F	F	NA
Age	7 yr	13 yr	8 yr	5 yr	10 mo	NA	NA	11yr	NA	TOP
Delection size (Mb)	0.96	NA	1.6	1.5	1.3–1.4	1.3–1.4	1.6	1.2	1.2	1.7
Deletion range	195519857- 196482211	NA	197174369- 198842531	(195777965–195778024) to (197310392–197310451)	NA	NA	197-199	197292774- 198466778	197292774- 198466778	194184392- 195887205
Inheritance	*De novo*	*De novo*	*De novo*	Maternal	Paternal	NA	NA	Maternal	NA	NA
Clinical features											
CHD	+	+	+	+	+	+	+	-	+	+
	VSD	patent foramen ovale	PDA	ASD	PDA, subvalvular aortic stenosis	PDA, pulmonic stenosis	VSD	-	pulmonic stenosis	VSD
developmental delay	+	+	+	+	NA	+	NA	+	NA	NA
mental retardation	+	+	-	+	NA	-	+	+	±	NA
Craniofacial dysmorphic features	+	+	+	+	+	NA	NA	+	NA	NA
	Microcephaly, full moon face, flattened facial profile, dental dystrophies, large ears, auricular polyp	Microcephaly, short philtrum, high nasal bridge, large posteriorly rotated ears, long narrow face, ocular abnormality	Central incisors, bilateral epicanthal folds	Microcephaly, hanging nasal columella, short philtrum, thin upper lip	Rotated ears, frontal bossing, prominent nose, long thin lip, short palpebral fissure, single palmar creases	NA	NA	Micrognathia, low set ears, widened nasal bridge, short philtrum, hypertelorism	NA	NA
Others	NA	hypospadias, horseshoe kidney	GE reflux, urinary dysfunction	malpositioned toes	GE reflux	NA	NA	NA	NA	NA

The canonical deletion region of 3q29 microdeletion syndrome almost ranges from 195.7 Mb to 197.3 Mb (Fig. 2), including more than 20 genes. Most reported 3q29 microdeletion cases included *PAK2* and *DLG1* genes, which are autosomal homologs of the X-linked genes *PAK3* and *DLG3*. Haplosufficiency of *PAK3* or *DLG3* was reported to be associated with mental retardation.^[16]^ Comparing our case with typical 3q29 microdeletion syndrome, our case presented an atypical 3q29 deletion with affected region ranging from 194.1 Mb to 195.8 Mb, consisting of 12 genes (Fig. 2).

*TFRC* (OMIM: 190010), as a morbid gene, encodes the transferrin receptor which is important and necessary for cellular iron, development of erythrocytes and the nervous system. It plays a critical role in intracellular iron transport. The mutation of *TFRC* was found to be associated with immunodefificiency 46 (OMIM: 616740).^[7]^ It could be abnormally overexpressed in epithelial ovarian cancer.^[19]^ Downregulation of *TFRC* is predictive of recurrent Major Depressive Disorder (MDD), which may indicate the role of the innate immune system in depression.^[20]^*XXYLT1* (OMIM: 614552), known as Xyloside α-1,3-xylosyltransferase, is a retaining Glycosyltransferase of the GT8 family. It can catalyze the addition of the second xylose to elongate the xylose-glucose disaccharide in the extracellular domain of Notch proteins. It was speculated that *XXYLT1* dysfunction due to 3q29 microdeletion might impair epidermal growth factor (EGF) xylosylation, leading to up-regulated Notch signaling.^[12,21]^ Human amplification of *XXYLT1* in several kinds of cancer is frequently associated with decreased Notch signaling.^[22]^*ACAP2* (OMIM: 607766), is an Arf-6 GTPase-activating protein which can inactivate Arf6 at the pericentrosomal endosomes and regulate neurite outgrowth.^[12]^ In addition, the inactivation or downregulation of *ACAP2* in human cells might contribute to cancer development.^[23]^*ATP13A3* (OMIM: 610232), containing18 exons, is a member of the P-type ATPase family of proteins. Its heterozygous mutation was associated with protein catalytic activity and the loss of *ATP13A3* mRNA expression might inhibit proliferation and cause apoptosis promotion in endothelial cells.^[24]^ Research on other genes involved are scarce. According to the ClinGen database, no supporting evidence of haploinsufficiency phenotypes for all 12 genes is available. Hence, further research is needed to define their implications and functions.

The utilization of chromosomal microarray analysis have facilitated the detection rate of subchromosomal imbalances, especially for prenatal cases with ultrasound anomalies. In order to avoid unnecessary abortions, the clinical significance of detected CNVs should be taken into account based upon deleted/duplicated size, genetic contents, inheritance pattern and clinical heterogeneity.^[2]^

## 5 Conclusions

In our study, we described a rare prenatal case with VSD, overlapping with part 3q29 microdeletion syndrome, which has not been reported before. For genetic disorders or syndromes of clinical significance, genetic counselling is offered. However, for chromosomal microscopic imbalances partially overlapping with the defined pathogenic syndrome, multiple factors, including deleted/duplicated size, genetic materials and phenotypic diversity, should be taken into consideration. As more similar 3q29 duplication cases and their associated phenotypes accumulate, it will help collect evidence on genotype-phenotype correlation of 3q29 microdeletion.

## Author contributions

**Conceptualization:** Fagui Yue, Ruizhi Liu.

**Data curation:** Qi Xi, Yuting Jiang.

**Formal analysis:** Shu Deng, Jing He.

**Funding acquisition:** Fagui Yue, Ruizhi Liu.

**Investigation:** Qi Xi.

**Methodology:** Shu Deng, Hongguo Zhang.

**Project administration:** Ruizhi Liu.

**Software:** Yuting Jiang, Jing He.

**Supervision:** Hongguo Zhang.

**Visualization:** Hongguo Zhang.

**Writing – original draft:** Fagui Yue.

**Writing – review and editing:** Ruizhi Liu.
